# End-Stage Renal Disease and Renal Replacement Therapy in older Patients

**DOI:** 10.5812/numonthly.1825

**Published:** 2012-03-01

**Authors:** Andrew Smyth

**Affiliations:** 1 Department of Nephrology, Galway University hospitals, Galway, Ireland; 2 Department of Medicine, National University of Ireland Galway, Galway, Ireland; 3 HRB Clinical Research Facility, National University of Ireland Galway, Galway, Ireland

**Keywords:** Chronic Kidney Disease, Peritoneal Dialysis, Aging

## Abstract

As the world’s population continues to age, practitioners encounter increasing numbers of older patients with end-stage renal disease (ESRD) who require renal replacement therapy (RRT). Conservative management may be considered in older patients and has been shown to offer comparable survival rates and hospital-free days to RRT patients. At present, for those who choose RRT, hemodialysis is the most commonly used modality. Many practitioners believe that peritoneal dialysis (PD), including assisted peritoneal dialysis, can be used safely in this population. Age is not a contra-indication to peritoneal dialysis, and a choice of modality should be offered to older patients. Assisted peritoneal dialysis has been used successfully in multiple regions without an increase in complication rates. Quality of life is an important issue for older patients with ESRD, and studies such as Broadening options for long-term Dialysis in the Elderly support the use of PD in older patients as it is associated with fewer fluctuations in symptoms of ESRD and less intrusion into people’s lives. This review discusses the appropriateness of initiating RRT in older patients, choices of modality, underutilization of PD in older patients, use of assisted PD, complication rates, and quality of life in these patients. overall, PD seems to be a safe and effective modality of RRT in older patients, and assisted PD can be used in patients with limited functional impairment.

## 1. Introduction

As the world’s population continues to age and the prevalence of both chronic kidney disease (CKD) (1) and end-stage renal disease (ESRD) are rising, nephrologists are increasingly faced with the decision to initiate renal replacement therapy (RRT) in older patients. Reports from the United States Renal Data System highlight that in patients age 70 years and older, the rates of Stage 3 and Stage 4 CKD have increased from 17.8% (1998–1994) to 37.8% (1999–2004) (2). Recent rates of incident dialysis patients mirror this, with 113 to 221 per million cases among 45- to 63-year-olds compared to 110 to 610 per million cases among 65- to 74-year-olds and 99 to 984 cases per million among people 75 years old and older (3).

## 2. Review Questions 

Given that the number older patients who may require RRT is increasing, several important issues need to be considered. This review will focus on modalities of RRT for older patients, why peritoneal dialysis (PD) is under-utilized in older patients, the role of assisted PD in select cases, complications from PD, the importance of quality of life (Qol) in older patients with ESRD, and the appropriateness of initiating RRT for ESRD.

## 3. Review Methods 

To deal with the abovementioned questions, an extensive literature search was performed using peritoneal dialysis, ageing, aging, elderly, and older population as keywords. Databases including PubMED and the Science Citation Index were reviewed from 1966 to the present day. As many of the research questions raised for this review do not have randomized-clinical-trial evidence to support decision making, all observational studies were included. Studies performed on animal models were excluded.

## 4. Appropriateness of RRT

As functional status may decline after the initiation of RRT, it is important to consider the timing of dialysis initation. An American study of nursing-home residents who began RRT showed a marked decline in functional status during the initiation of dialysis, and at 1 year there was a significant decline in functional capacity from the predialysis level (4). More recently, there has been a dramatic increase in the frequency of patients initiating RRT with estimated glomerular filtration rates (eGFR) above 10 ml/min/1.73m^2^, often referred to as an “early start.” In 1996, 15% of patients initiated RRT with an eGFR of 10 to 14.9 ml/min/1.73m^2^, and 4% initiated RRT with an eGFR greater than 15 ml/min/1.73m^2^. In 2005, 30% started dialysis with an eGFR of 10 to 14.9 ml/min/1.73m^2^, and 15% initiated dialysis with an eGFR greater than 15 ml/min/1.73m^2^. A large increase was also seen in patients over the age of 75 at the time of initiating RRT, with the frequency of early starts rising from 25% in 1996 to 54% in 2005 (5).

It has been hypothesized that an increase in preemptive dialysis (in those with weight loss, anorexia, and malnutrition) contributes significantly to this. Early starts may impact residual kidney function (RKF), which has been associated with better nutritional status (6). Interestingly, one study reported patients over the age of 75 had a slower rate of RKF decline without initiating RRT, and patients in the CKD Stage 4 cohort of this study were more likely to die than to develop ESRD (7). This raises questions about the appropriateness of initiating RRT in these patients at all. When initially approached about RRT, a significant number of older patients often decide not to commence RRT. This may reflect cultural differences, such as the belief that one has “lived long enough.” Counseling should be provided in those instances to ensure that patients make a truly informed choice regarding RRT (8). Conservative management may be a better choice to maintain Qol. Non-dialysis therapy provided by a multidisciplinary nephrology team can offer comparable survival rates without the risks and impact on Qol from RRT (9). Many believe that the emotional investment of patients and health care professionals in life-prolonging RRT in some patients may cause unnecessary death or medicalization, including invasive testing, procedures, and hospitalizations with only marginal benefit (10). Carson et al. recently demonstrated that, on average, dialysis prolongs survival for elderly patients with ESRD with significant comorbidity by 2 years, but that patients who chose conservative management can survive for a substantial length of time while achieving a similar number of hospital-free days (11). Ideally, patients with advancing CKD should be evaluated by a multidisciplinary team including geriatric specialists to assess the medical, social, and family environment prior to any decision about PD, hD, or conservative treatment (12). 

## 5. Which RRT Modality Is Best? 

No consensus exists as to the optimum modality of RRT in older age groups. Significantly, hemodialysis (HD) and PD patients have been shown to have similar life expectancy and expected years of life lost, with the exception of patients with diabetes, who have better outcomes with HD (13). HD is often thought to be unsuitable for older patients due to risks of hypotension and arrhythmia (14), and as such, many nephrologists believe PD to be a suitable alternative. PD may be advantageous as it is a continuous home-based therapy with better hemodynamic stability, steady-state metabolic control, and hypertension control (15). Because it is a home-based therapy, the potential for disrupting patient–family relationships is low, and the need for potentially expensive and uncomfortable transport to and from a dialysis unit multiple times per week is avoided (16).

In practice, older patients are less likely to start PD than younger patients, even in countries and regions with a high rate of PD utilization (16). Many practitioners argue that age is a barrier to the initiation of PD due to increased comorbidity. older patients are more likely to have physiological decline of organ function, which may subsequently influence the course and outcome of ESRD treatment (17). In contrast, a recent French study found that age was not important in the 6-month prognosis in older patients initiating RRT, but low BMI, diabetes, heart failure, peripheral vascular disease, dysrhythmia, malignancy, and behavior disorders were more important predictive factors. Functional dependency and unplanned initiation of dialysis were also associated with increased mortality (18). Most practitioners believe that patients should have a free choice of modality (PD or HD) (19) and that practitioners underutilize PD as a modality in older patients (20). In summary, based on the available evidence, the best practice is to offer a free choice of RRT modality to older patients and their partners and family members.

## 6. Why PD Is Underutilized in Older Patients 

Many practitioners believe that age is a barrier to the initiation of any form of RRT (21) because older age has been associated with an increased risk of mortality (22). other practitioners believe that more important factors than age alone are late referral to nephrology (sometimes called crash landers), social isolation, and the level of functional dependence (23). Because most patient education is provided by doctors and nurses in nephrology units, any bias these health care providers may have against PD can greatly influence patient choice of modality; in fact, not all modalities are available in all regions due to physician preference (16). A recent review argues that low rates of PD utilization may be due to a systematic failure to educate patients on the process of care (24) and that with appropriate education and support from the multidisciplinary team, over 50% of older patients may be eligible for and choose PD (21, 25). As such, older patients with advancing CKD ideally should be provided with unbiased (26) information regarding the availability of RRT modalities, advantages, contra-indications, and impact of treatments on outcomes and Qol, preferably several months before the commencement of dialysis (27).

This is particularly noticeable in the United States, where the overall rate of PD utilization is low. A recent study found this to be the case despite PD being discussed as an option in 61% of cases, with only 10.9% deciding to initiate PD instead of hD (28). This problem is evident in many other countries as well. For instance, in Europe, where PD utilization rates are higher, elderly patients remain less likely to utilize PD than hD (29). The Netherlands Cooperative Study on the Adequacy of Dialysis offered patients a choice of dialysis modality, although approximately one-third of patients were deemed to have medical or social contraindications to a particular modality. of the remaining two thirds, factors associated with choosing hD over PD included being over age 70, being of female gender, and living alone. It is important to note that those who had attended predialysis education were more likely to choose PD (21). This highlights the importance of education and provides patients with adequate information to make an informed choice prior to the onset of ESRD and being able to plan initiation of RRT. Based on the available evidence, age alone should not be considered a barrier to the initiation of RRT, particularly PD, in older patients. Given that functional dependence is increasingly prevalent in older populations, alternative forms of RRT, including assisted PD, should also be considered.

## 7. Assisted PD 

older patients with ESRD may have significant comorbidity, impaired vision, deafness, poor mobility, arthritis, and cognitive decline, as well as to be socially isolated, live in poor accommodations, or have financial problems. These issues may impair functional ability, and independent PD may not be possible. It has been estimated that 61.2% of patients over the age of 80 years requires assistance with dialysis exchanges, exit-site care, and medications (30). Another study highlighted that three quarters of PD patients over the age of 65 years required some assistance from either family members or caregivers (31). These issues may also arise in patients who previously performed PD independently and lost functional independence for several reasons. In such cases, assisted PD (AsPD) may be a viable option (20, 32).

The utility of AsPD is internationally recognized, and a number of countries provide dedicated funding to facilitate AsPD. A Chinese study reported AsPD to be a good option for patients with limited functional ability (33). In the Republic of Ireland, where there is no dedicated funding available to support AsPD, the treatment has been successfully employed (without any difference in PD outcomes) when there is adequate spousal or family support (34). Because AsPD provided by the family or spouse is often performed by one dedicated person, it is thought that complication rates such as exit-site infection may be similar to those utilizing independent PD. Interestingly, a Danish study reported AsPD to be feasible, safe, and efficient in patients with an unplanned start of RRT immediately after PD catheter insertion (35). Assisted PD can safely be used in older patients with limited functional ability or independence with no significant increase in complication rates or technique survival. AsPD has been used successfully in programs where there is dedicated funding to facilitate health-care provided AsPD and also in regions without funding, where spouses or family members provide the assistance.

## 8. Complications 

The most common complications of PD include peritonitis and exit-site infection, which impact morbidity and technique failure rates. It is often hypothesized that older patients are at increased infection risk due to immunodeficiency of aging and malnutrition. our review of the literature shows that complication rates vary significantly. For example, our recent paper failed to show any change in overall survival or complication rates in older age groups (34). A recent Australian study reported that older patients have higher peritonitis-related and all-cause mortality rates but similar rates of peritonitis-free survival and superior-technique survival (36). Another study concluded that exit-site and tunnel infections may be less frequent in the elderly on PD because they are less active than younger patients and may disturb the exit-site dressings and tubing less frequently (37). Infection-related hospitalizations are frequent in older patients using either RRT modality, including many unrelated to dialysis access. It is important to note that this outcome does not appear to differ significantly between PD and hD patients (38).

Early mortality rates (within 90 days of initiation of RRT) remain high for all RRT patients, but the rate is even higher (27%) in the elderly ESRD population. Mortality rates within a year of initiating RRT among patients over 70 are reported to be as high as 35%. The rate increases to over 50% in those age 80 or older (39). This is often related to comorbidities, but an important factor is late referral to nephrology services (40). This is particularly important, as the initiation of RRT may be delayed due to non-nephrologists’ overestimations of GFR due to the effect of diminished muscle mass on serum creatinine. late RRT starts are associated with longer initial hospitalizations and increased frequency and duration of subsequent hospital admissions (41). A recent Japanese study reported that cardiac performance at initiation of PD therapy is predictive of the prognosis in patients over 75 (42). our recent study reported no difference between younger and old patients who initiated PD in terms of overall survival or peritonitis-free survival but also showed a trend toward longer hospital stays in older patients (34). Although reported rates vary, overall the rates of complications or mortality do not appear to be higher in older patients who chose to perform PD (including AsPD) over hD.

## 9. Quality of Life 

Qol is an important issue for all patients with ESRD, but particularly in older patients who often do not have the opportunity for renal transplantation. Depression is common in all patients receiving RRT but has been cited as being even more prevalent in older patients (43), with particularly high rates in women (44). Functional status (ability to use the bathroom, bathe, dress, etc.) is a key aspect of Qol and has been shown to be a strong predictor of survival (45). high rates of impaired functional status have been reported in patients with ESRD, (46) but a recent study reported that there is no difference in cognitive and motor functioning in stable hD and PD patients (47).

The majority of studies of Qol in ESRD patients focus on the population as a whole, and few have focused specifically on the elderly, with the exception of the North Thames Dialysis Study. This study reported that a relatively high proportion of elderly patients were being treated with PD and that the outcomes of survival and Qol were not different between PD and hD patients (48). Still, providing patients with a free choice of modality of dialysis has been shown to improve Qol (49), and the principal determinants of Qol in older people are the value of being independent and in control of day-to-day living (50). older patients generally prefer to receive dialysis at home via PD or home hD when possible (51). A Turkish study compared health-related Qol, sleep quality, and depression scales in automated PD (APD) and CAPD patients and found no significant differences in any of these outcomes by treatment method (52).

As randomized controlled trials comparing outcomes and Qol between hD and PD patients are not feasible (because it is not possible to randomize patients to one modality over another), observational studies remain the only option to compare treatment methods. The recently published Broadening options for long-term Dialysis in the Elderly study adds greatly to the literature. This multicenter, UK-based study, aimed to enable a higher proportion of older patients to receive the modality of their choice and compared matched PD and hD patients. The study supports the use of PD in older patients by showing similar Qol in both groups, with PD being favored over hD with fewer symptoms and less intrusion into older people’s lives (16). overall, older patients experience less intrusion with home-based RRT, including both APD and CAPD. The initiation of RRT impacts significantly on older patient’s Qol.

## 10. Conclusion 

Renal replacement therapy, including peritoneal dialysis, is a potential treatment modality in older patients with ESRD. A choice of modality should be offered to all patients with ESRD, including older patients, as age alone should not be considered a barrier. Assisted PD is an option in patients with functional impairment, as complication rates are not higher in this population. It is important to consider the timing of RRT initiation, with respect to eGFR and residual kidney function, and the subsequent impact on Qol. home-based therapies offer older patients who chose RRT the best potential Qol.
